# Laparoscopic onlay-flap ureteroplasty using cecal appendix

**DOI:** 10.1590/S1677-5538.IBJU.2023.0595

**Published:** 2024-03-18

**Authors:** Miquel Amer-Mestre, Valenti Tubau, Ricardo Guldris-García, Javier Brugarolas Rossello, Enrique Pieras Ayala

**Affiliations:** 1 Son Espases University Hospital Department of Urology Palma Balearic Islands Spain Department of Urology, Son Espases University Hospital, Palma, Balearic Islands, Spain.

## Introduction:

The management of ureteral strictures longer than 1-2 cm must be treated by major surgery (1, 2). The strictures located at the distal part of the ureter can be managed by a ureteral reimplantation using a psoas hitch or a Boari flap depending on its proximity to the bladder (3). Those located at the proximal ureter can be treated by a pyeloplasty (4). The ureteric strictures in the mid-ureter are the ones that pose a greater challenge for the urologist because a ureteral substitution is needed, either using a segment of the intestine or a buccal mucosa graft (5, 6). Our main objective is to present the management and results at 36 months of a patient with a right mid-ureter stricture.

## Material and methods:

A 63-year-old male with chronic kidney disease (CKD) and a right single functioning kidney was referred to our department with the diagnosis of a 3 cm stricture in the right mid-ureter. He had a long-term JJ-stent in place but in the last year we had to replace it three times precociously and he even needed the placement of a nephrostomy tube due to the obstruction of the JJ-stent. Accordingly, a permanent resolution was sought and a laparoscopic onlay-flap ureteroplasty using cecal appendix was performed.

## Results:

The first step was to identify the cecal appendix. Then we identified and dissected the ureter. With the ureter dissected, we performed a ureteroscopy to pinpoint the stricture. Once we knew where the stricture was, we proceeded with the ureterotomy and preparation of the cecal appendix. The final step was to perform the ureteroplasty between the ureter and the cecal appendix placing a JJ-stent before the last stitches were done. Total operative time was 190 minutes without any intraoperative complication. The JJ-stent was removed 7 weeks later. The follow-up of the patient was done with regular blood test and ultrasound to rule out deterioration of the CKD and worsening of the residual hydronephrosis. With a follow-up of 36 months, the patient is stent free, he hasn't had any further intervention and neither the CKD nor the hydronephrosis haven't worsened.

## Conclusions:

Laparoscopic onlay-flap ureteroplasty using cecal appendix is a feasible and well tolerated procedure for patients with right mid-ureter stricture. However, we must bear in mind the difficulty of these cases and they should be performed in expert centers.
